# Supplementary material for: ScnR1-Mediated Competitive DNA Binding and Feedback Inhibition Regulate Guvermectin Biosynthesis in Streptomyces caniferus
Source: Biology (Basel). 2025 Jul 4;14(7):813. doi: 10.3390/biology14070813 (PMC12292694; doi:10.3390/biology14070813)
Supplement: Supplementary file 1 [file biology-14-00813-s001.zip › biology-3705635-supplementary.pdf]

Supplementary Materials

ScnR1-Mediated Competitive DNA Binding and Feedback Inhibition Regulate Guvermectin Biosynthesis in *Streptomyces caniferus*

Haoran Shi, Jiabin Wang, Xuedong Zhang, Na Zhou, Xiangjing Wang, Wensheng Xiang, Shanshan Li and Yanyan Zhang

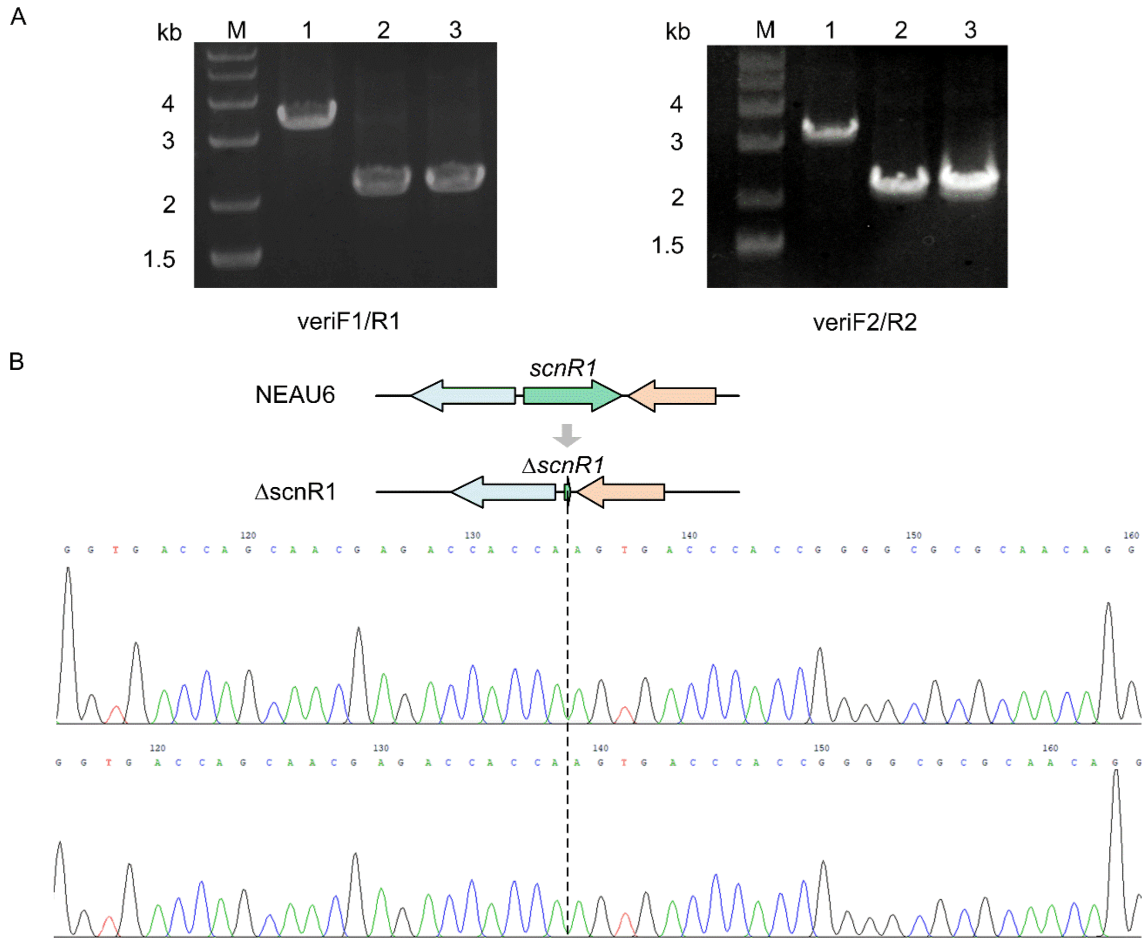

Figure S1. Confirmation of *scnR1* deletion by PCR amplification (A) and DNA sequencing (B). PCR templates were genomic DNAs from NEAU6 (lane 1) and the two independent *scnR1* deletion mutants as indicated (lanes 2–3). The primer pairs used are also shown.

Table S1. Strains and plasmids used in this work

Name	Description	Source
**Strains**		
Escherichia coli		
JM109	General cloning host for plasmid manipulation	Novagen
ET12567(pUZ8002)	Donor strain for conjugation between *E. coli* and *Streptomyces*	[1]
BL21(DE3)	Host for protein expression	Novagen
Streptomyces caniferus NEAU6	Parental strain for guvermectin production	[2]
ΔgvmR	*gvmR* inactivation strain	[3]
NEAU6/gvmR	NEAU6 containing pSET152::gvmR	[3]
NEAU6/P_{hrdB}gvmR2	NEAU6 containing pSET152::P_{hrdB}gvmR2	[3]
NEAU6/P_{hrdB}scnR1	NEAU6 containing pSET152::P_{hrdB}scnR1	This work
ΔscnR1	*scnR1* deletion strain	This work
NEAU6/pSET152	NEAU6 containing pSET152	This work
**Plasmids**		
pKC1139	Apr^r, integrative *E. coli-Streptomyces* shuttle vector for gene disruption	[4]
pKC1139::scnR1	For in-frame deletion of the *scnR1* gene, generated from pKC1139	This work
pSET152	Integrative *E. coli-Streptomyces* shuttle vector	[1]
pSET152::P_{hrdB}	pSET152 containing the *hrdB* promoter	Laboratory stock
pSET152::P_{hrdB}scnR1	pSET152 containing one copy of *scnR1* driven by the *hrdB* promoter	This work
pGEX4T-1	Vector for GST-tagged protein expression in *E. coli*	GE Healthcare
pGEX-4T-1::scnR1	ScnR1 expression vector based on pGEX-4T-1	This work
pGEX-4T-1::gvmR	GvmR expression vector based on pGEX-4T-1	[3]
pGEX-4T-1::gvmR2	GvmR2 expression vector based on pGEX-4T-1	[3]

Table S2. Primers used in this work

Primers	Sequence (5'-3') [1]	Usage
**For gene overexpression and deletion**		
HscnR1-F	**TTTTCAACGTTCCGAGAGGTTGTTC**GTGACCAGCAACGAGACCACCAC	Overexpression of *scnR1*
HscnR1-R	**TGCCAAGCTTGGGCTGCAGGTCGACTCTA**GGCGCTTCGGGCGAGGAC	Overexpression of *scnR1*
ΔscnR1-RF	**TAAAACGACGGCCAGTGCCAAGCTT**TTCGTCGCGGTATTGATGAT	For amplification of the right homologous arm of *scnR1*
ΔscnR1-RR	TGGTGGTCTCGTTGCTGGTC	
ΔscnR1-LF	**CAGGTGACCAGCAACGAGACCACCA**AGTGACCCACCGGGGCGCG	For amplification of the left homologous arm of *scnR1*
ΔscnR1-LF	**CGCGCGCGGCCGCGGATCCTCTAGA**CGGGCCCGAGGCATGTGGAT	
veriF1	GCCCCGACCTTCAGCATCAT	For the verification of knockout strains
veriR1	CTGCTGGACGAGAGCGGCG	
veriF2	ATACGTATCATGACCCCCACCCCAG	
veriR2	TCAAGCGCCACTACGGCCTCA	
**For qRT-PCR**		
real-16s-F	TGTCGTGAGATGTTGGGTTAAG	Control
real-16s-R	TCATTGTACCGGCCATTGTAG	
real-R-F	TGGGTTATCGGCTCAATCTTTC	Transcription analysis
real-R-R	TGTGCACATCGGCGAAAT	
real-A-F	ATGAACGCAAGGCACTGA	Transcription analysis
real-A-R	CATGTCAGGACCTCGAACAG	
real-E-F	TGATCGCCCGCAAGAAG	Transcription analysis
real-E-R	TCCTCACCGAGGAAGAGTT	
real-R1-F	GACAGTTGACCACCCATCTG	Transcription analysis
real-R1-R	AGGTCGAAGGTCCAGGAG	
real-R2-F	GTCTGCGATGACGACAACAT	Transcription analysis
real-R2-R	GCATCGTTGAAGCCGATCA	

**For protein expression**		
GEXscnR1-F	**GGTTCCGCGTGGATCCCCGGAATTC**GTGACCAGCAACGAGACCACCAC	Overexpression of ScnR1 in *E.coli*
GEXscnR1-R	**GTCAGTCACGATGCGGCCGCTCGAG**CTCCCCGCCGGGGCGCG	
**For EMSAs**		
gvmRA-pF	CCACTTTCCCGCCATCACCAA	Probe P_{R-A}
gvmRA-pR	CGTACCGCACGAGGCTCTGC	
gvmT1-pF	GATGGTGTACGCGATGATGATGC	Probe P_{T1}
gvmT1-pR	ACGAAGGCGACCAGCAGGAA	
gvmT2-pF	GGCGTAGCCGGAGACCAGGTA	Probe P_{T2}
gvmT2-pR	GGTCATCTGCGACGACGACAATCT	
gvmE-pF	GTGATGAGCGTCGAACCGGG	Probe P_{E}
gvmE-pR	AGTTCCACGTCGCCGTACAGCT	
gvmF-pF	AACATGGTCAAGCCGCTCCG	Probe P_{F}
gvmF-pR	ACGGCGTAACGCTCGCGTAT	
1133/34-pF	GCTTGTGACAGTGCCTTGAA	Probe $P_{1133-1134}$
1133/34-pR	GGTGAGGATGGCGTATCG	
1543/44-pF	ATTGCAGACGCCGTAGACCG	Probe $P_{1544-1543}$
1543/44-pR	TCGCCCAGGACGAGCGAGAC	
0026-pF	GCGTCGCGGAGTTCGAGGTGAA	Probe P_{0026}
0026-pR	ACAGCGGCCCTGCCTCTGTT	
0174-pF	GGCCACTCCCGGACATCA	Probe P_{0174}
0174-pR	GGCCGGTCGTTCTTGGAT	
0741-pF	GACCCCAGCAGTCACCACA	Probe P_{0741}
0741-pR	CCACGACCCAGAAGAACCAG	
1209-pF	TCGCCGAGCATCTCCTGT	Probe P_{1209}
1209-pR	GTTCATTGACCCGCACCC	

1410-pF	GATTTCCTTGACGGTGGCG	Probe P_{1410}
1410-pR	GGACAGCAGGGCTTGTTCG	
1744-pF	ACCGCCTGCGCCGAAGTGAT	Probe P_{1744}
1744-pR	CCCGTGCGTGGCTGGAAAT	
1780-pF	AACCCAGTTGCCGAGGACA	Probe P_{1780}
1780-pR	TCCACAGCACGACGGTCAG	
1876-pF	GGTCGCCAGCACCAGCCA	Probe P_{1876}
1876-pR	CTGGAAGAGAAGGTAGGCGGCC	
1920-pF	GGCAATCTTCCCGGCGCGC	Probe P_{1920}
1920-pR	GGGTGCTCCTCAGCGATGCC	
1939-pF	GGCGACACCACCAACACCA	Probe P_{1939}
1939-pR	ATGAGCTGGACAATTCCGTAGA	
1991-pF	CGATCAGCTCCACGCCCGGATA	Probe P_{1991}
1991-pR	GGCGACGAAACGAGGCGC	
2100-pF	GAGCTGGCAGGTCAGAGAGCT	Probe P_{2100}
2100-pR	GGCTCCAGGTGGCCAGCCA	
2106-pF	GGAAGCCCACCACGTTGACGAT	Probe P_{2106}
2106-pR	ACCAGTTCGTCCGGGCGG	
2320-pF	TCACCGCATCCCGCCAGG	Probe P_{2320}
2320-pR	CCGTCAGCTCGTGCTCCAC	
2888-pF	GCCCCACCCCTGCTGTGA	Probe P_{2888}
2888-pR	TGCGGTCGAGTCGTCTGC	
2961-pF	AAGTGCTCGGTGGGAACATGC	Probe P_{2961}
2961-pR	TCGGCGATGATCCAGCGGT	
3087-pF	TGGTGATCTCGATGAACTCTGG	Probe P_{3087}
3087-pR	CCGTCCGAATCCTGGTGC	

3181-pF	ACAGGCCTACGTACCCACC	Probe P_{3181}
3181-pR	GCACTTCGCAGTGCGGCA	
3187-pF	CACTCCCACCGTGACAAAGA	Probe P_{3187}
3187-pR	GACCAGCGAAAAGGACAAAA	
3479-pF	ACTGGGTGATCTTCGCCTGGC	Probe P_{3479}
3479-pR	CCAGGGCGAGGTCTCCGT	
3524-pF	GCGATGCCGAACGGGATG	Probe P_{3524}
3524-pR	CGAAATCGGCACCCAGCA	
3535-pF	TAGCGGACCTGGGTGTGCG	Probe P_{3535}
3535-pR	TGCGCAAGGCCACCGAGC	
3567-pF	AGCATCGCCGTCAGGTAGTACTT	Probe P_{3567}
3567-pR	GGTGAGGAAGCCACCGGTCAC	
3624-pF	CTGATCGGTGGGGAAGTGGAGG	Probe P_{3624}
3624-pR	ACCCGCGTGAAGAGACCGG	
3682-pF	ACCCGGAGTCCGATATGAGGC	Probe P_{3682}
3682-pR	CGGTAGGCGAGAAGGAAGGAAA	
3696-pF	CACGGTGCTGCAGCGCAT	Probe P_{3696}
3696-pR	CCCTGCTGAGCTGAGGCGAC	
3709-pF	GGACGTCGGTGGAGAGGACG	Probe P_{3709}
3709-pR	TGGTCCCCCGCCCTCGAC	
3712-pF	CGTTGACCCCGTGCAATTC	Probe P_{3712}
3712-pR	CCAGTCACCGAGCGGAAAG	
4030-pF	GCGGTGCCGTTCGGTGTG	Probe P_{4030}
4030-pR	CACCAGAGCGGCGAGGGAGA	
4384-pF	GCACACCCGTCAGCAGCTC	Probe P_{4384}
4384-pR	ACGCGACGGTGTAGGCGAT	

4507-pF	GTCGAGACGGCCGAGTTCCC	Probe P_{4507}
4507-pR	GGCGATGGAGCCCACGGAA	
4595-pF	CGGGTCGGTGCTGTCTGC	Probe P_{4595}
4595-pR	CGATCACCTCCAGGAGCG	
4691-pF	AGGCCACGGTGGCCGTCA	Probe P_{4691}
4691-pR	CAGAAAGCCGCCCAGCAG	
4714-pF	CGGTCCGAGTCGGGGGAG	Probe P_{4714}
4714-pR	GACGGCCACGATCTCGGC	
4782-pF	GTGCGCAGGGTGTGCTGCA	Probe P_{4782}
4782-pR	GCACCGTCCGTACCCGGAT	
4948-pF	CTAACCGGTTCAGCCCCTCGTA	Probe P_{4948}
4948-pR	TCCCGCAGCTCCCGGACG	
4997-pF	GATTTCGGCGGTGGTGCT	Probe P_{4997}
4997-pR	GCGGGCTGATCGGTCATA	
5103-pF	AGCTCACGCTGCACCAGC	Probe P_{5103}
5103-pR	CGCATTTCCGCCACCATC	
5223-pF	TGGGGCTGCTGCTGTACACG	Probe P_{5223}
5223-pR	TTCTTGCCGAGCGCGTCGT	
5227-pF	CGCGTCGTGATCCGCCTTG	Probe P_{5277}
5227-pR	CGATCTCGCGCTCTTCCAGGT	
5331-pF	ACCTGCTCGGTGACGGACTG	Probe P_{5311}
5331-pR	GTTGACGATGCCCTTGGTG	
5381-pF	CCTGGAGGCCAGGGACGG	Probe P_{5381}
5381-pR	CGAGGAGGGCTTGAGCTCGAT	
5700-pF	GGTCTGGGCGATCTGCAC	Probe P_{5700}
5700-pR	GGTCTCCCCGAGCTTGTCA	

5778-pF	TCGTCGAGGACTTCACCAGC	Probe P_{5778}
5778-pR	GAGAAACGTGGCGAGGGA	
5806-pF	GACGGCACGTCGGGTGCG	Probe P_{5806}
5806-pR	TGGACGGCAGGTATGGCGG	
5926-pF	CTCGCCGCTGGGTATGTT	Probe P_{5926}
5926-pR	TGCCCGCCAAGTGAAGTG	
6201-pF	ACGAGGTGGATGTCGGTGTC	Probe P_{6201}
6201-pR	CGTGCCAGGTGCGGTAGAA	
6257-pF	CGGCGTCCATGTATCCGG	Probe P_{6257}
6257-pR	GGAGTTGGGCCCGTCATC	
6438-pF	CCTGCCAGTGACGGGAGCCT	Probe P_{6438}
6438-pR	GCCCAGCCGGTGGAACAA	
6724-pF	CTTCGTGGTCCCCGGAGAGC	Probe P_{6724}
6724-pR	GCAGCAGCTGCTGGACCT	
6774-pF	GTCGGTGCCGTGTTCCAT	Probe P_{6774}
6774-pR	TGAGCAGTGCCTCGTGCA	
6992-pF	CAGGACGCGCTCGTAATCGG	Probe P_{6992}
6992-pR	TCCACGACACGAGGCACACG	
7577-pF	CATGGTGTTCGTGGTGAGC	Probe P_{7577}
7577-pR	CGATCGGGACCAGCATCT	

[1] Bold type characters indicate the homologous fragments

Table S3. Genes whose promoter regions contain sequences similar to GvmR binding sites

#	Locus tag	Function	sequence [1]
1	*NEAU6-1GM001133*	hypothetical protein	gtcatacgcatgac
2	*NEAU6-1GM001134*	isochorismatase	gtcattcgtatgac
3	*NEAU6-1GM001543*	MFS transporter	gtgatacgtataac
4	*NEAU6-1GM001544*	LacI family transcriptional regulator	gttatacgtatcat
5	*NEAU6-1GM004948*	*methyltransferase*	atcctacgtttgaa
6	*NEAU6-1GM005806*	KsbA	atcctccgtatgct
7	*NEAU6-1GM000174*	major facilitator transporter	gccgtacgcgccac
8	*NEAU6-1GM006257*	monooxygenase	ctcatacgtcggat
9	*NEAU6-1GM003087*	hypothetical protein	gtcacgcggatgac
10	*NEAU6-1GM004691*	hypothetical protein	atcatcggcatcgc
11	*NEAU6-1GM000026*	hypothetical protein	gggatacggatgac
12	*NEAU6-1GM001744*	hypothetical protein	gtcatacgcctcgc
13	*NEAU6-1GM003187*	transglycosylase	gtcacacgactgac
14	*NEAU6-1GM000741*	amino acid transporter	gtgatgcggctcac
15	*NEAU6-1GM003682*	peptidase	gttgtccgcatcac
16	*NEAU6-1GM005381*	branched-chain amino acid aminotransferase	gttcaacggatcgt
17	*NEAU6-1GM005926*	hypothetical protein	ctgatgcgtgtgat
18	*NEAU6-1GM005103*	membrane protein	gtgattcgactgac
19	*NEAU6-1GM001939*	ABC transporter permease	gacatacgaacgac
20	*NEAU6-1GM006438*	hypothetical protein, partial	gtgatgcgaaacat
21	*NEAU6-1GM006201*	PucR family transcriptional regulator	aagatcctcatctc
22	*NEAU6-1GM003624*	AraC family transcriptional regulator	atgtcttgcatgtc
23	*NEAU6-1GM006724*	hypothetical protein, partial	gtccgccgcaccac

24	*NEAU6-1GM003181*	NADH dehydrogenase	attgttcgtccaac
25	*NEAU6-1GM001410*	preprotein translocase subunit YajC	atccttcgcacgac
26	*NEAU6-1GM004997*	1D-myo-inositol 2-acetamido-2-deoxy-alpha-D-glucopyranoside deacetylase	gctgtcggtatgac
27	*NEAU6-1GM005331*	ABC transporter substrate-binding protein	atcatgn_{32}catcac
28	*NEAU6-1GM007577*	hypothetical protein	gagatgcgtatgac
29	*NEAU6-1GM005700*	DNA-binding protein	ggatgacn_{20}gtcattc
30	*NEAU6-1GM004595*	hypothetical protein	gcatcacn_{10}gtcatcg
31	*NEAU6-1GM002320*	hypothetical protein	gaatgacn_{87}gtcatcc
32	*NEAU6-1GM002888*	sugar ABC transporter substrate-binding protein	gtaccacn_{49}gtatcac
33	*NEAU6-1GM001920*	helicase	gagtgacn_{3}atcactc
34	*NEAU6-1GM005778*	PTS ascorbate transporter subunit IIC	gccatacn_{163}gtacaac
35	*NEAU6-1GM001780*	signal peptidase	gcatgacn_{17}gtgatgc
36	*NEAU6-1GM006774*	hypothetical protein	ctgattcn60gtcatgc
37	*NEAU6-1GM003524*	membrane protein	atgatgcn_{93}gcatcgg
38	*NEAU6-1GM001209*	membrane protein	gtggtacn7gtggtac
39	*NEAU6-1GM003567*	MULTISPECIES: NADH dehydrogenase subunit A	ggatgagn_{534}ctcatac
40	*NEAU6-1GM003535*	MULTISPECIES: NADH-ubiquinone oxidoreductase subunit 3	ggatgggn_{129}ggatcgg
41	*NEAU6-1GM004384*	NADH-quinone oxidoreductase subunit D	gggatgcn_{45}ccgatgc
42	*NEAU6-1GM001991*	succinate dehydrogenase	gatgagcn_{16}ctgatgg
43	*NEAU6-1GM004782*	succinate dehydrogenase	gtgtgatn_{20}gtgtgat
44	*NEAU6-1GM002100*	cystathionine beta-lyase	ggattacn_{67}ggactac
45	*NEAU6-1GM002106*	cytochrome C oxidase subunit II	ccttatan_{196}tttatgc
46	*NEAU6-1GM001876*	protoheme IX farnesyltransferase	ggtcatcn_{99}ctgatgt
47	*NEAU6-1GM004030*	ABC transporter	tcatcacn_{28}gtgatgc
48	*NEAU6-1GM005227*	ATP synthase F0F1 subunit alpha	gatccggn_{8}gatccgg

49	*NEAU6-1GM005223*	ATP synthase F0F1 subunit A	ccgatgcn_{107}cccatgc
50	*NEAU6-1GM004507*	polyphosphate kinase	ctgatacn_{16}ctgatac
51	*NEAU6-1GM003712*	phosphoribosylamine--glycine ligase	gcatgcan_{45}gtcatcc
52	*NEAU6-1GM003696*	phosphoribosylaminoimidazole synthetase	atgacggn_{229}atcatgc
53	*NEAU6-1GM002961*	phosphoribosylaminoimidazole carboxylase	gtcatacn_{91}ggatgat
54	*NEAU6-1GM003709*	phosphoribosylaminoimidazole-succinocarboxamide synthase	ttgatccn_{10}tcgattc
55	*NEAU6-1GM006992*	adenylosuccinate lyase	gcatgagn_{32}gcatgtg
56	*NEAU6-1GM004714*	Phosphoribosylaminoimidazole-carboxamide formyltransferase	gcatgagn_{1}gcatcaa
57	*NEAU6-1GM003479*	adenylate kinase, partial	gcatcagn_{81}aggatgc

[1] The space length between each pair of inverted repeats is indicated by red color font.

Table S4. Comparison of retention times, peak areas, and guvermectin production

Sample	Retention time (min)	Guvermectin peak area (mAU)	Guvermectin production (mg/L)
Guvermectin standard	9.2	1522.8	75.1
NEAU6	9.2	2496.2	646.5
NEAU6/pSET152	9.2	1988.3	510.1
ΔscnR1	9.2	2588.1	671.1
pSET152::P_{hrdB}scnR1	9.2	0	0

Table S5. EMSA grayscale analysis of ScnR1 binding affinity to wild-type and mutant $P_{R\text{-}A}$ probes

Lane	Free Probe	Upper band	Lower band
$P_{R\text{-}A}$ (ScnR1)			
0	100	0	0
0.05	100	0	0
0.1	70.3	9.9	19.8
0.2	30.6	29.1	40.3
0.3	3.6	70.3	26.1
$P_{R\text{-}A}$-M1 (ScnR1)			
0	100	0	0
0.05	100	0	0
0.1	73.7	9.7	16.6
0.2	51.2	18.0	30.8
0.3	20.0	37.6	42.4
$P_{R\text{-}A}$-M2 (ScnR1)			
0	100	0	0
0.05	100	0	0
0.1	71.8	10.0	18.2
0.2	51.2	18.1	30.7
0.3	3.2	49.5	47.3
$P_{R\text{-}A}$-M3 (ScnR1)			
0	100	0	0
0.05	100	0	0
0.1	100	0	0
0.2	81.0	9.4	9.6
0.3	61.1	20.8	18.1

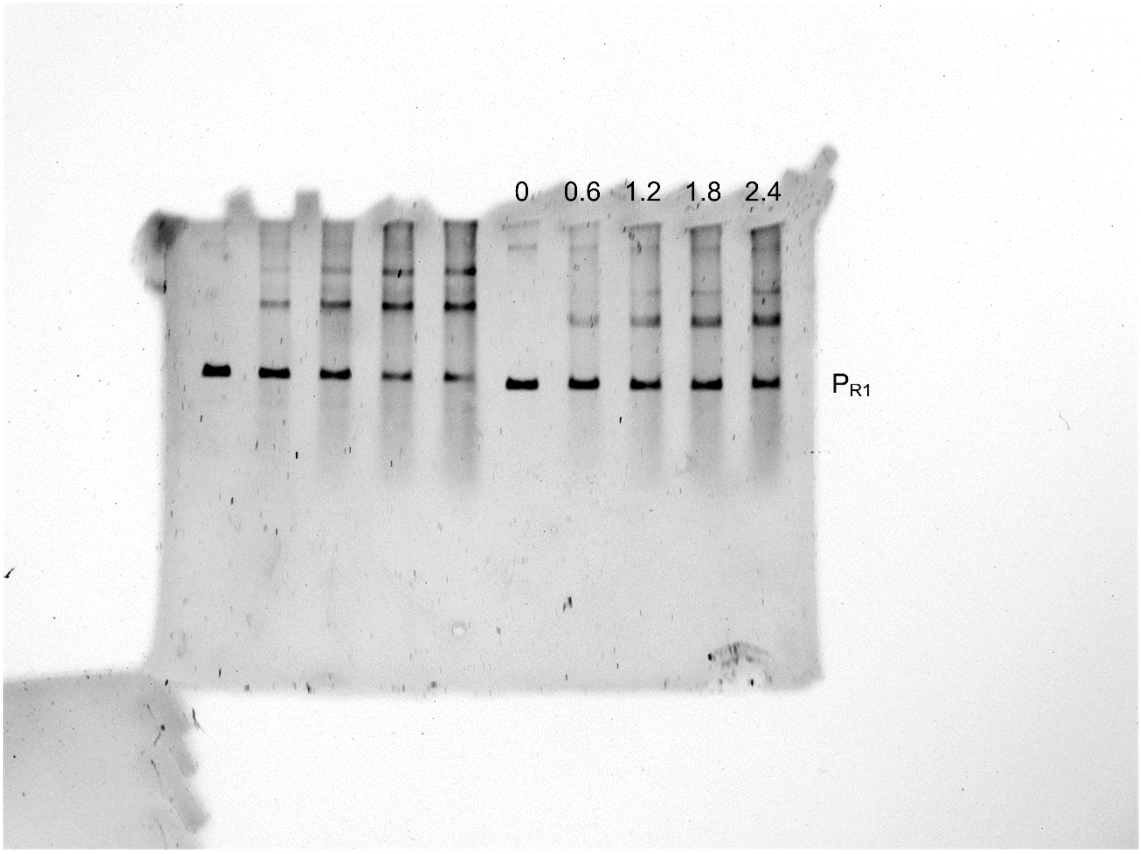

Figure S2. Original image of Figure 1B.

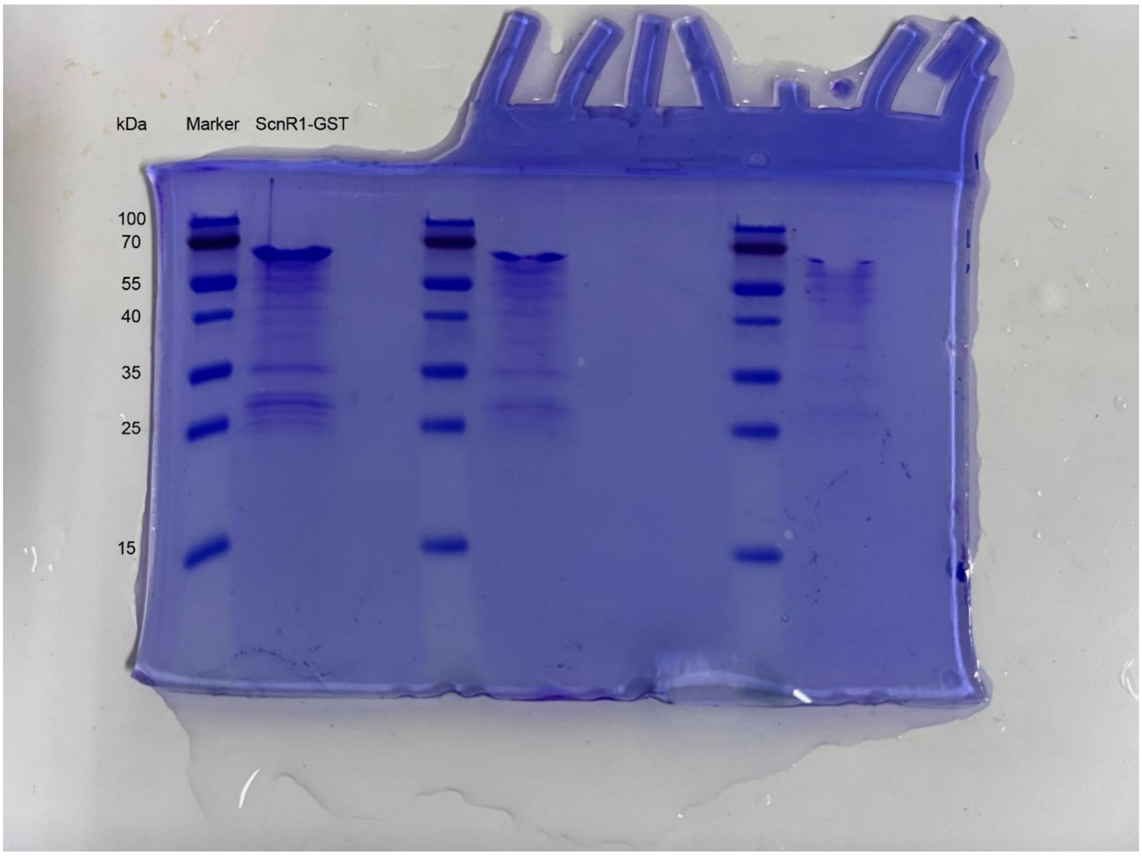

Figure S3. Original image of Figure 3B.

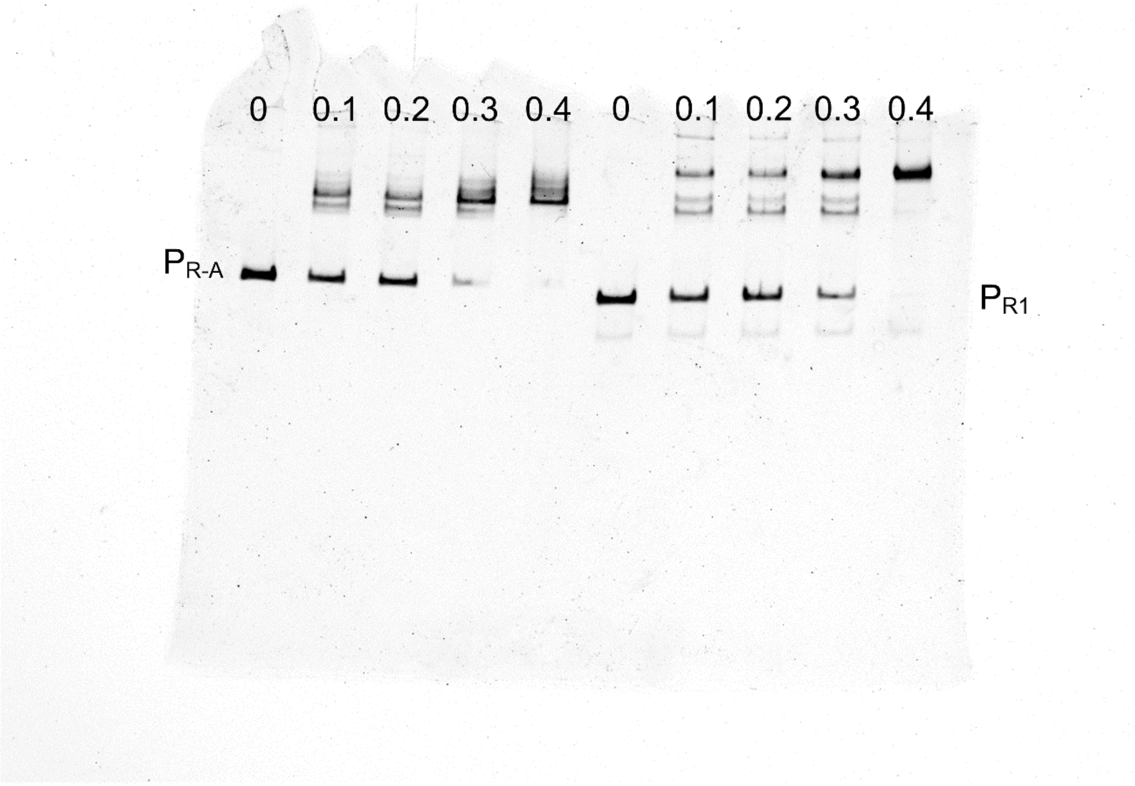

Figure S4. Original image of Figure 3C,E.

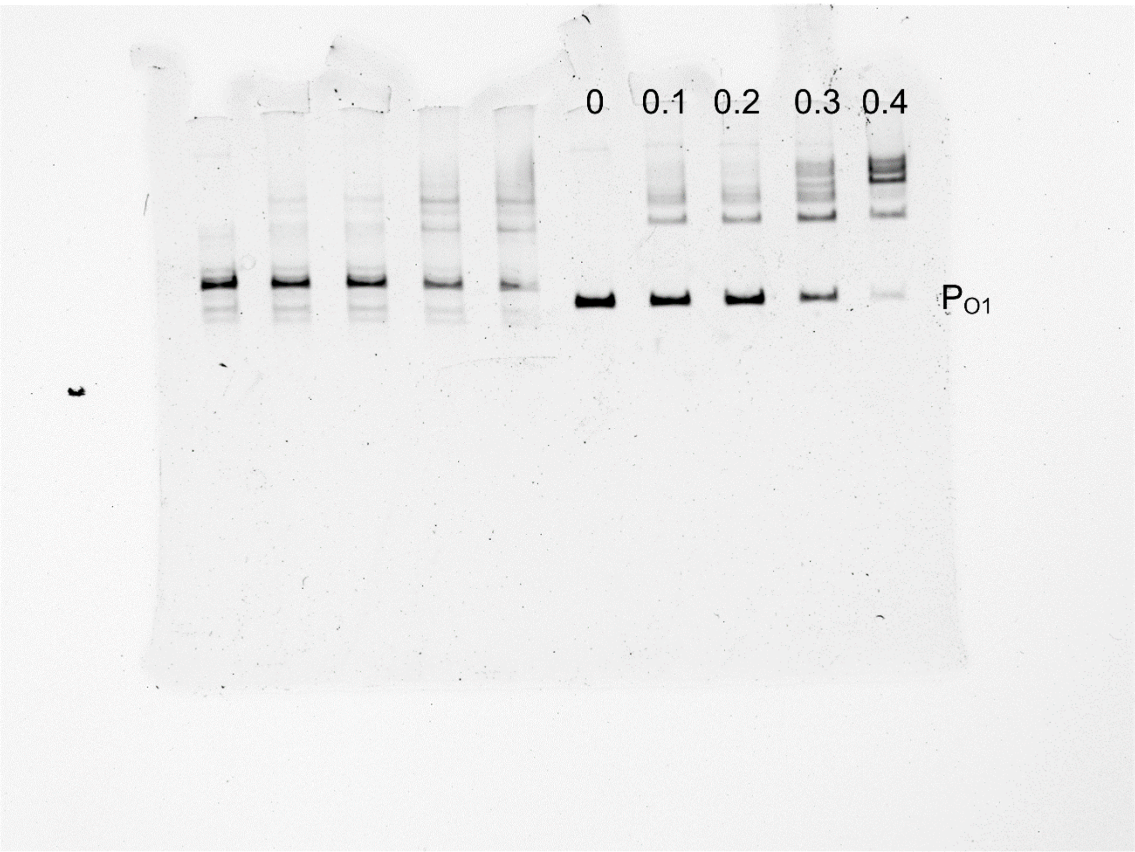

Figure S5. Original image of Figure 3D.

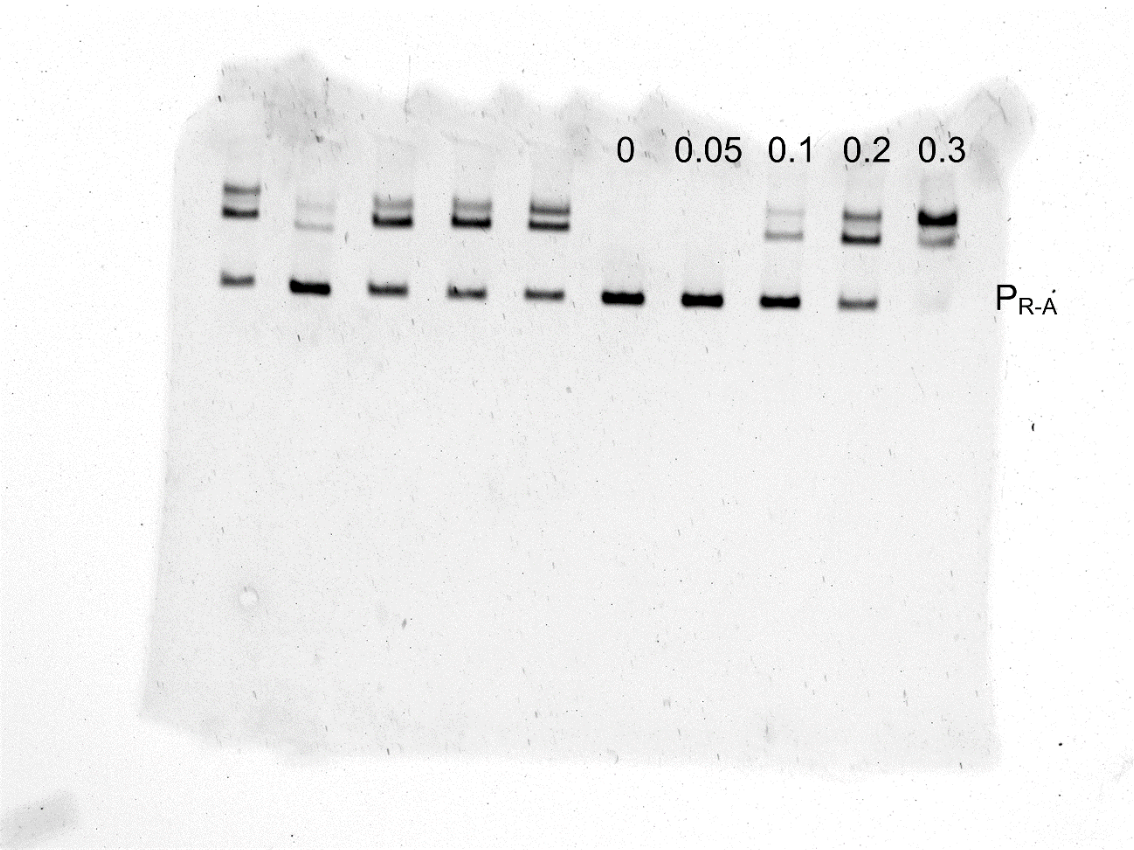

Figure S6. Original image of Figure 4D-P(R-A).

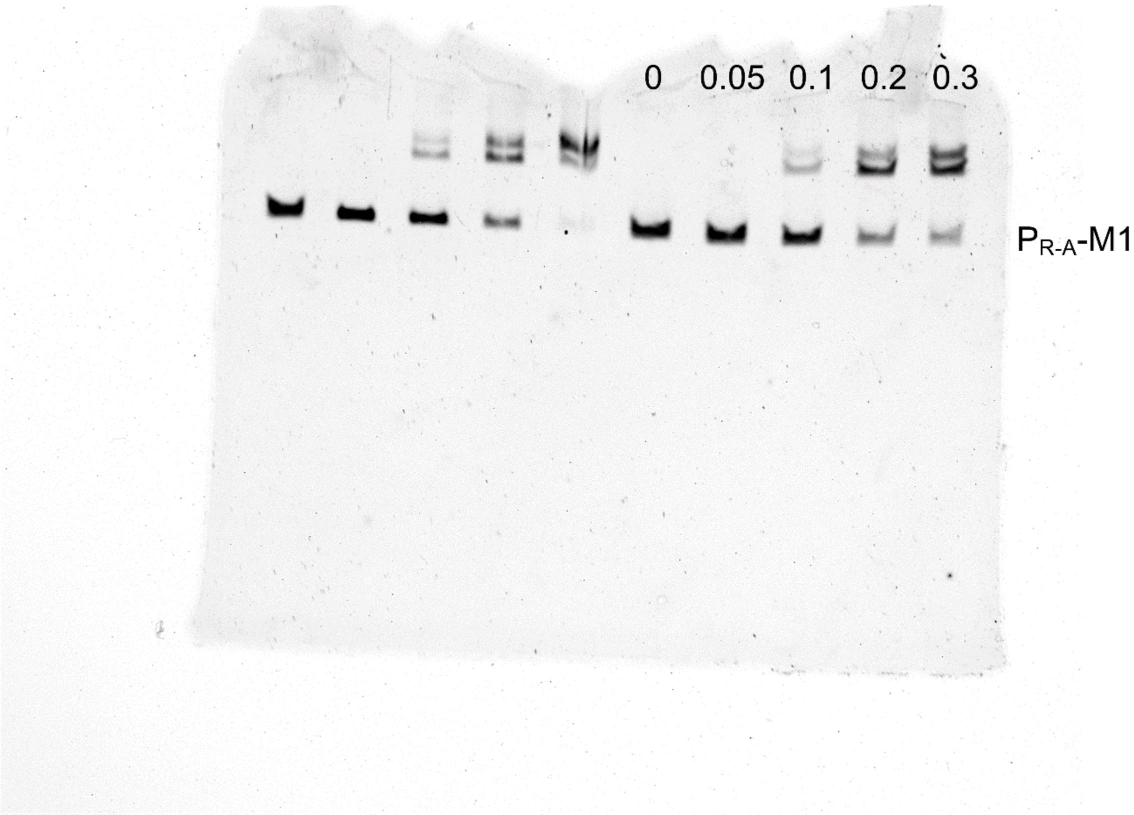

Figure S7. Original image of Figure 4D-P(R-A)-M1.

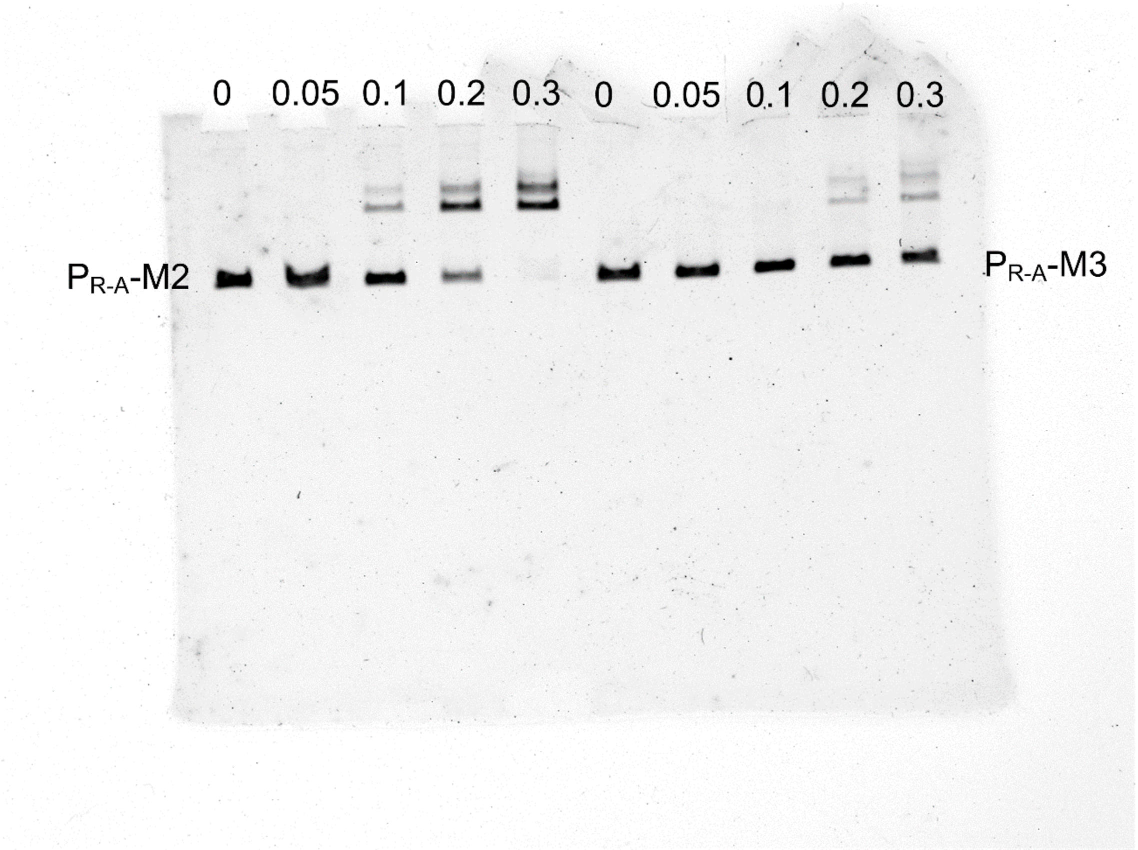

Figure S8. Original image of Figure 4D-P(R-A)-M2M3.

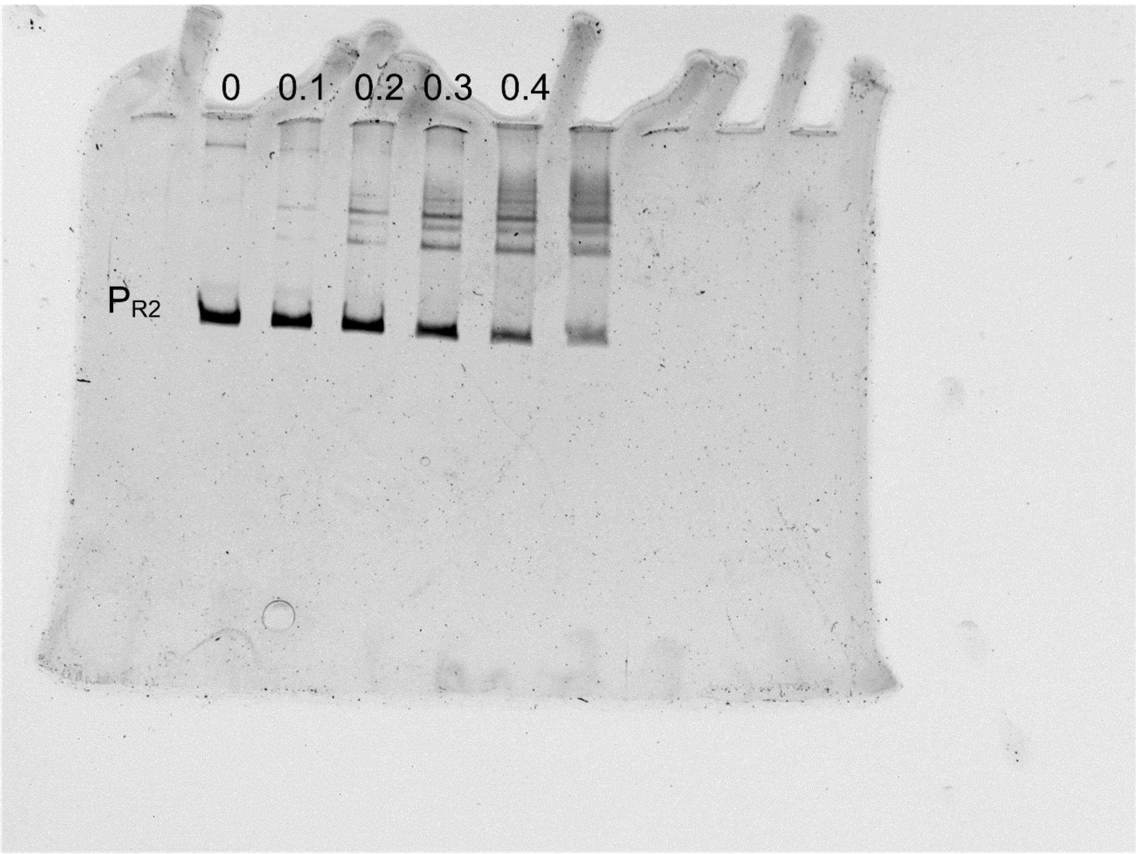

Figure S9. Original image of Figure 5A-P(R2).

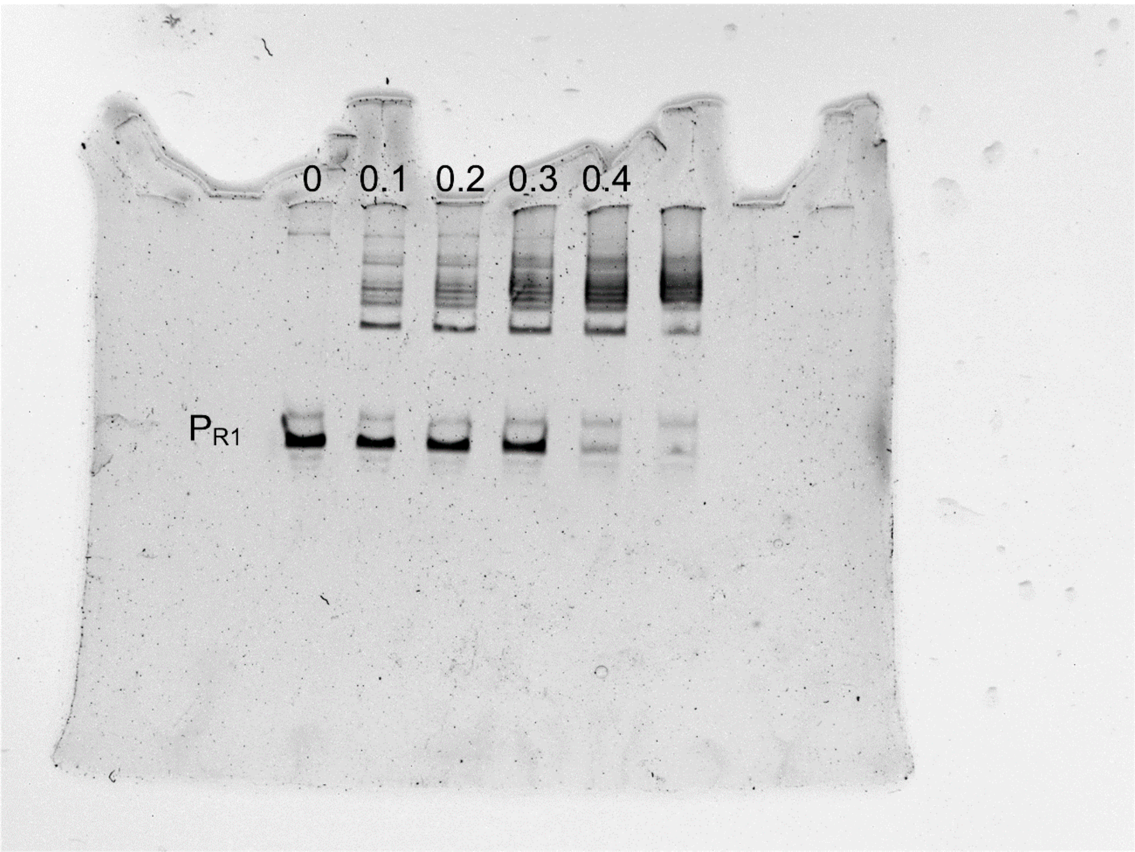

Figure S10. Original image of Figure 5A-P(R1).

References

1. Kieser, T.; Bibb, M.J.; Buttner, M.J.; Chater, K.F.; Hopwood, D.A. *Practical Streptomyces Genetics*; John Innes Found: Norwich, UK, 2000; Volume 291.
2. Liu, C.; Wang, Z.; Chen, Y.; Yan, Y.; Li, L.; Wang, Y.J.; Bai, L.; Li, S.; Zhang, Y.; Wang, X.; et al. Guvermectin Biosynthesis Revealing the Key Role of a Phosphoribohydrolase and Structural Insight into the Active Glutamate of a Noncanonical Adenine Phosphoribosyltransferase. *ACS Chem. Biol.* **2023**, *18*, 102–111.
3. Shi, H.; Wang, J.; Li, S.; Liu, C.; Li, L.; Dong, Z.; Ye, L.; Wang, X.; Zhang, Y.; Xiang, W. Coordinated regulation of two LacI family regulators, GvmR and GvmR2, on guvermectin production in *Streptomyces caniferus*. *Synth. Syst. Biotechnol.* **2025**, *10*, 237–246.
4. Bierman, M.; Logan, R.; O'Brien, K.; Seno, E.T.; Rao, R.N.; Schoner, B.E. Plasmid cloning vectors for the conjugal transfer of DNA from *Escherichia coli* to *Streptomyces* spp. *Gene* **1992**, *116*, 43–49.
